# Rational Design of Peptide Binders Targeting Prominin‑1 and Sortilin for Molecular Sensing Applications

**DOI:** 10.1021/acsomega.6c01105

**Published:** 2026-07-01

**Authors:** Samavath Mallawarachchi, Nirmitee Mulgaonkar, Samuel Mabbott, Shreya A. Raghavan, Sandun Fernando

**Affiliations:** 1 Department of Biological and Agricultural Engineering, 14736 Texas A&M University, College Station, Texas 77843, United States; 2 Department of Biomedical Engineering, 14736 Texas A&M University, College Station, Texas 77843, United States

## Abstract

Prominin-1 (CD133) and sortilin are two clinically important biomarkers that have been associated with multiple types of cancer. Designing molecules targeting these biomarkers is highly useful for both detection and targeted treatment of cancer. Current detection strategies for these biomarkers primarily rely on receptor-specific antibodies. However, antibodies have several limitations, including high sensitivity to glycosylation in prominin-1, poor tumor penetration, and limited suitability for a broad range of biomolecular assays. These limitations highlight the need for alternative targeting molecules, such as peptides. This study employs a peptide design approach based on residue-level binding for the rational design of peptides targeting prominin-1 and sortilin. This approach is based on identifying the amino acids with the highest predicted affinities toward the receptor and combining the strongest binding amino acids to create peptide sequences. Initially, candidate peptides with docking scores less than −8 kcal/mol were screened using molecular docking, and the binding stability of these peptides was verified by using molecular dynamics simulations. Preliminary experimental validation via biolayer interferometry indicated that these peptides bind to prominin-1 and sortilin with micromolar-level affinities. While the binding behavior of sortilin-targeting peptides suggested some heterogeneous binding, the overall binding behavior was consistent with micromolar-level affinities. This approach provides a promising method for developing peptide binders targeting critical biomarkers, such as prominin-1 and sortilin.

## Graphical abstract

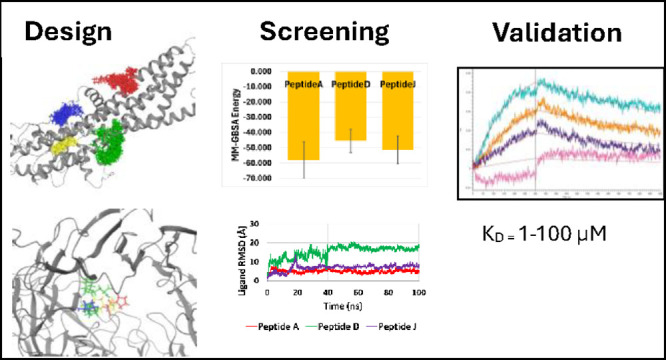

## Introduction

Detecting and targeting cancer biomarkers is a key research focus in modern oncology.^1^ This study presents a novel in silico technique to design peptide binders targeting prominin-1 and sortilin, two medically significant biomarkers. Prominin-1, also known as CD133, has been identified as a common biomarker for cancer stem cells (CSCs) for many cancer types, including gastric, breast, skin, lung, colon, ovarian, and pancreatic cancers, and is frequently used for isolation of cancer cells.^2,3^ It is a transmembrane glycoprotein consisting of five transmembrane domains, two large extracellular loops, and two small intracellular loops.^4^ Prominin-1 is reported to be a regulator of signaling pathways associated with cell proliferation, differentiation, migration, intercellular communication, and apoptosis, and is believed to play a key role in tumorigenesis, metastasis, and chemoresistance of CSCs.^3,5^ The critical role of prominin-1 in signaling pathways and its biomarker characteristics make it a highly important receptor for the detection and treatment of cancer.^3^

Currently, the detection of prominin-1 is mainly done using fluorescent spectrometry and flow cytometry. Recently, surface plasmon resonance has been reported as a label-free technique for the quantification of prominin-1.^6^ Current approaches for the detection of prominin-1 and targeted drug delivery to prominin-1-containing cells require prominin-specific monoclonal antibodies.^2^ However, due to the presence of multiple *N*-glycan structures in prominin-1, antibody binding is highly sensitive to glycosylation modifications, which vary between different cell types, that can lead to inconsistencies in detection.^4,7–9^ Also, most of the currently available antibodies are only suitable for specific types of biological assays.^10^ Therefore, other molecule types such as prominin-specific aptamers and peptides have been studied as substitutes for antibodies in prominin-1 detection. Compared to antibodies, these aptamers and peptides reported in literature have shown several advantages, such as lower glycosylation sensitivity, better tumor penetration, less immunogenicity, and easier synthesis.^4,11–13^

Sortilin, also known as neurotensin receptor-3 (NTR3), is a membrane protein belonging to the vacuolar protein sorting 10 protein (Vps10p) family of sorting receptors.^14^ Sortilin has the capacity to bind to more than 50 different proteins, making it a versatile receptor that is vital to many cellular processes. The primary function of sortilin is sorting and transporting proteins between the Golgi apparatus, endosomes, and lysosomes.^15^ In addition, it is also involved in neuronal signaling and immune response modulation.^16,17^ Sortilin is reported to be a biomarker for multiple types of cancer, including brain, liver, skin, breast, and pancreatic cancers, as well as cardiovascular disease and diabetes mellitus.^14,18,19^ In a study done on glioblastoma multiforme patients, it has been found that higher sortilin expression was associated with a lower chance of survival, and inhibition of sortilin can reduce the invasiveness of cancer cells.^14^ Sortilin knockdown in breast cancer cells has been reported to inhibit the migration and invasion of cancer cells.^20^ Anti-sortilin antibodies have also shown the ability to cause apoptosis of leukemic cells in leukemia patients.^21^ This suggests that sortilin is a suitable therapeutic target, as well as a biomarker.

Detection of sortilin is currently done using sortilin-specific antibodies, using fluorescent spectrometry and flow cytometry.^21^ However, it is reported that some of the commercially available antibodies have failed to detect sortilin in some cell types, indicating lower reliability for diagnostic applications.^21^ Therefore, there is an urgent need for new molecules that can effectively target sortilin. While the use of sortilin-targeting peptides for biosensors has not been reported yet, they have been successfully used to inhibit sortilin-mediated degradation of progranulin.^22^ Therefore, the development of new peptides targeting sortilin offers promising opportunities for biosensing and therapeutic applications.

In this study, we apply a peptide design approach based on residue-level binding to design peptides targeting prominin-1 and sortilin. By computationally optimizing peptide structures, this approach has the potential to design new peptides that can be used for the detection and targeting of critical biomarkers, with the goal of overcoming the challenges in current antibody-based approaches.

## Materials and Methods

### Protein and Ligand Structures

The protein structure for prominin-1 was developed using AlphaFold,^23^ based on the protein sequence obtained from the UniProt database (UniProt ID: O43490). The structure for sortilin was obtained from RCSB PDB (PDB ID: 5NMT).^24^ Amino acid structures were obtained from the ZINC15 database.^25^ The terminal carboxyl groups of amino acids were replaced with less reactive carbonyl groups to reduce terminal reactivity and emulate the behavior of amino acids in peptide bonds.^26^ LigPrep was used to generate the most probable protonation states of the amino acids at pH 7.0. Peptide structures were developed using the Peptide Builder tool in Schrödinger 2021–3. All protein and peptide structures were optimized and minimized prior to docking using the Schrödinger Protein Preparation Wizard.

### Molecular Docking

Amino acid probes and peptides were docked on the receptor proteins using Schrödinger Glide 2021–3, and the amino acids and peptides with the highest predicted affinities were identified based on docking scores and Glide binding energies. Since the active site of prominin-1 is not elucidated in the literature, the SiteMap module in Schrodinger was used to identify the potential active sites. The docking grid for prominin-1 was centered around the residues LEU11 and ALA774 to incorporate the best two sites predicted by SiteMap, and the grid size was maintained at 36 Å. For sortilin, docking was done at the neurotensin binding active site reported in literature, using ARG292, PHE317, TYR318, SER319, and ILE320 as the grid center.^24^

### Molecular Dynamics Simulations

Molecular dynamics (MD) simulations were conducted using Schrödinger Desmond. Protein–ligand complexes from the best docking conformations were solvated using the TIP3P water model, and the system was neutralized by adding Na^+^ or Cl^–^ ions. Since prominin-1 contains transmembrane domains, a membrane using the POPC model was placed, selecting the residues 109–129, 158–178, 434–454, 487–507, and 793–813 as the transmembrane regions based on UniProt data. MD simulations were conducted for 100 ns with a recording interval of 20 ps, using the NPγT ensemble at 300 K, 1.01325 bar, and 0 surface tension. Post simulation analysis, including root mean square deviation (RMSD) and root mean square fluctuation (RMSF), was conducted using the simulation interaction analysis tool.

Trajectory MM-GBSA values were calculated using the thermal_mmgbsa.py script, based on 250 frames during the last 50 ns of the simulation. Entropic contributions to the binding were calculated using the following interaction entropy (*T*Δ*S*) equation.^27,28^

$$T\Delta S=-RT\ln\;<e^{\left(\frac{\Delta E_{int}-<\Delta E_{int}>}{RT}\right)}>$$

where Δ*E*_int_ = Δ*E*_Coulomb_ + Δ*E*_van der Waals_

### Materials

HIS-tagged human sortilin protein (UniProt ID: Q99523) was purchased from Acro Biosystems, and His-tagged recombinant human prominin-1 protein was purchased from US Biological Life Sciences. Peptides targeting prominin and sortilin were custom-synthesized by Biomatik based on the sequences provided by the authors. Anti-Penta-HIS (HIS1K) biosensors were purchased from Sartorius. The 10X kinetics buffer (1X PBS pH7.4, 0.02% Tween 20, 0.1% BSA) was prepared and filter-sterilized for the BLI experiments.

### Biolayer Interferometry

Biolayer interferometry (BLI) was conducted using Sartorius Octet R4 to analyze the binding kinetics of the designed peptides on prominin and sortilin. After an initial baseline step of 60 s, the receptor protein was loaded on HIS1K biosensors, followed by a second baseline step of 60 s. Loading concentrations of 5 and 10 μg/mL were used for prominin and sortilin, respectively. Association (300 s) and dissociation (600 s) profiles for each peptide were measured at four concentrations. A double referencing scheme was used during the data processing. A 0 μM analyte concentration (buffer only) was used as a reference sample to correct the baseline drift. Unloaded biosensors were used as reference sensors at each concentration to eliminate the impact of non-specific binding. The final binding curves were obtained by subtracting the signals from the reference biosensors and zero concentration wells. The kinetic parameters were calculated with Sartorius Octet Analysis Studio Software using the 1:1 global-fitting model. The assay was carried out in triplicate, and kinetic parameters were expressed as mean ± standard deviation.

## Results and Discussion

### Peptide Design Targeting Prominin-1 (CD133)

Since the exact active site of prominin-1 has not been elucidated yet, we used SiteMap to predict the most probable druggable sites. The sites predicted by SiteMap are illustrated in Supporting Information Figure S1, and the properties of the sites are included in Table S1. As shown in Figure 1, the top three predicted druggable sites were located in close proximity to each other. Site 1 exhibited the most favorable properties, with a site score of 1.179 and a druggability (*D*) score of 1.295 (scores greater than 1 usually indicate druggable sites). It also had a sufficient site volume and a favorable enclosure value of 0.751. Site 2 also exhibited similar properties with a smaller volume. These two sites mainly included residues in the ranges of MET1-GLY12, LEU80-TYR105, TYR412-LEU427, ASN510-PHE556, and ALA774-LEU783, which are located in the extracellular loops and thus easier to target.^29^ Since sites 1–3 are adjacent high-ranking sites, the docking grid was selected to encompass these three sites.

**Figure 1:**
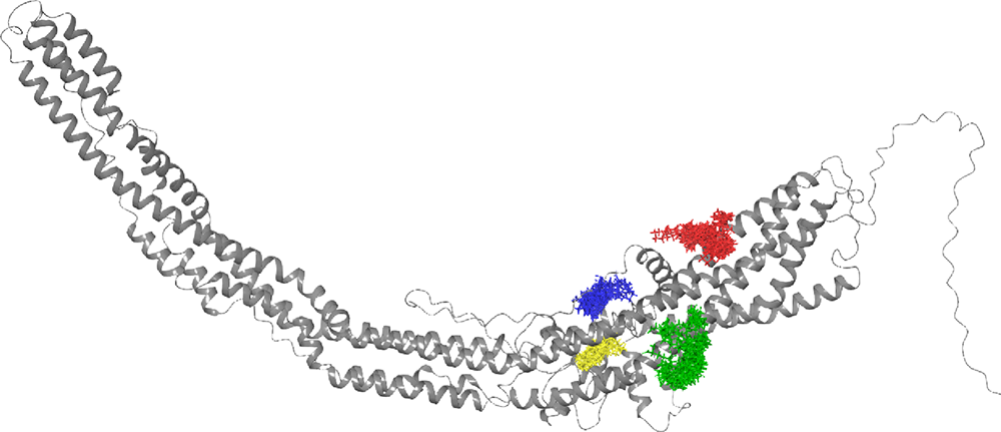
Amino acid binding sites in the predicted druggable region of prominin-1: site 1 (yellow), site 2 (green), site 3 (red), and site 4 (blue).

As the initial step of the peptide design process, amino acid probes were docked at the predicted druggable sites of prominin-1 identified by SiteMap. It could be observed that the amino acids bound to four closely located locations on prominin-1. The predicted amino acid binding sites are depicted in Figure 1, and docking scores and binding energies of the amino acids demonstrating the most favorable binding at each site are given in Table 1.

**Table 1:** Docking Scores and Glide Energies of the Strongest Binding Amino Acid Probes at Each Site in Prominin-1^a^

site	amino acid	docking score (kcal/mol)	Glide energy (kcal/mol)
1	TRP	–6.209	–19.907
1	TYR	–6.036	–21.998
1	PHE	–5.913	–18.117
2	HIS	–5.591	–13.027
2	ARG	–4.381	–19.776
2	PHE	–3.968	–13.405
3	TRP	–4.023	–13.916
3	PHE	–3.821	–13.176
3	PRO	–3.776	–13.490
4	HIS	–6.470	–20.646
4	ARG	–5.999	–27.630
4	TRP	–4.950	–21.876

a All values are expressed as the average of the three best conformations.

As shown in Table 1, TRP and HIS showed favorable binding to most of the sites, suggesting a potentially high affinity to prominin-1. In general, the amino acids demonstrated stronger binding to sites 1 and 4, which are closer to the center of prominin-1, suggesting that the peptides are more likely to bind to this region. It could be observed that amino acids binding to both sites 1 and 4 interacted with the ALA774-ASP780 region. Therefore, the peptides were docked into this region using ASP776 as the grid center.

Ten peptides were designed by using combinations of the strongest binding amino acids in each of these clusters and linking these residues using short peptide linkers. Two types of linkers were used in this study: linkers based on amino acids showing the most favorable binding, and conventional peptide linkers GGS and GSG.^30^ These peptides were docked on prominin-1, and overall, the peptides demonstrated favorable binding, with an average docking score of −8.593 ± 0.343 kcal/mol, and an average Glide energy of −75.213 ± 6.096 kcal/mol. Six peptides exhibited docking scores lower than −8.5 kcal/mol and Glide energies lower than −70 kcal/mol. Three peptides were screened from that group based on docking scores, binding energies, and interactions with prominin-1. The docking results of the three selected peptides are given in Table 2, and the binding conformations of peptides and the interactions of the peptides with prominin-1 are shown in Figure 3. Docking information for all peptides is included in Table S2 in the Supporting Information.

**Table 2:** Docking Scores and Binding Energies of Selected Peptides on Prominin-1^a^

peptide	sequence	docking score (kcal/mol)	Glide energy (kcal/mol)	MM-GBSA energy (kcal/mol)
Peptide A	WGSGHGSGH	–8.527	–84.700	–34.963
Peptide D	HGGSHGGSW	–8.508	–81.450	–28.200
Peptide J	PWHRWHRWYF	–8.939	–71.476	–42.047

a All values are expressed as the average of the three best conformations.

As illustrated in Figure 2a, all three peptides bound to approximately the same region in prominin-1, spanning from site 1 to site 4. According to Table 2, all three peptides showed docking scores of less than −8.5 kcal/mol and glide energies of less than −70 kcal/mol, suggesting potentially strong binding.^31^ However, average MM-GBSA energies were in the range of −25 to −45 kcal/mol, indicating that the binding is weaker in a solvated environment, which could be due to the hydrophobic nature of the binding pocket. Peptide J showed the most negative MM-GBSA energies, suggesting that it could have the most favorable binding under a solvated environment.

**Figure 2:**
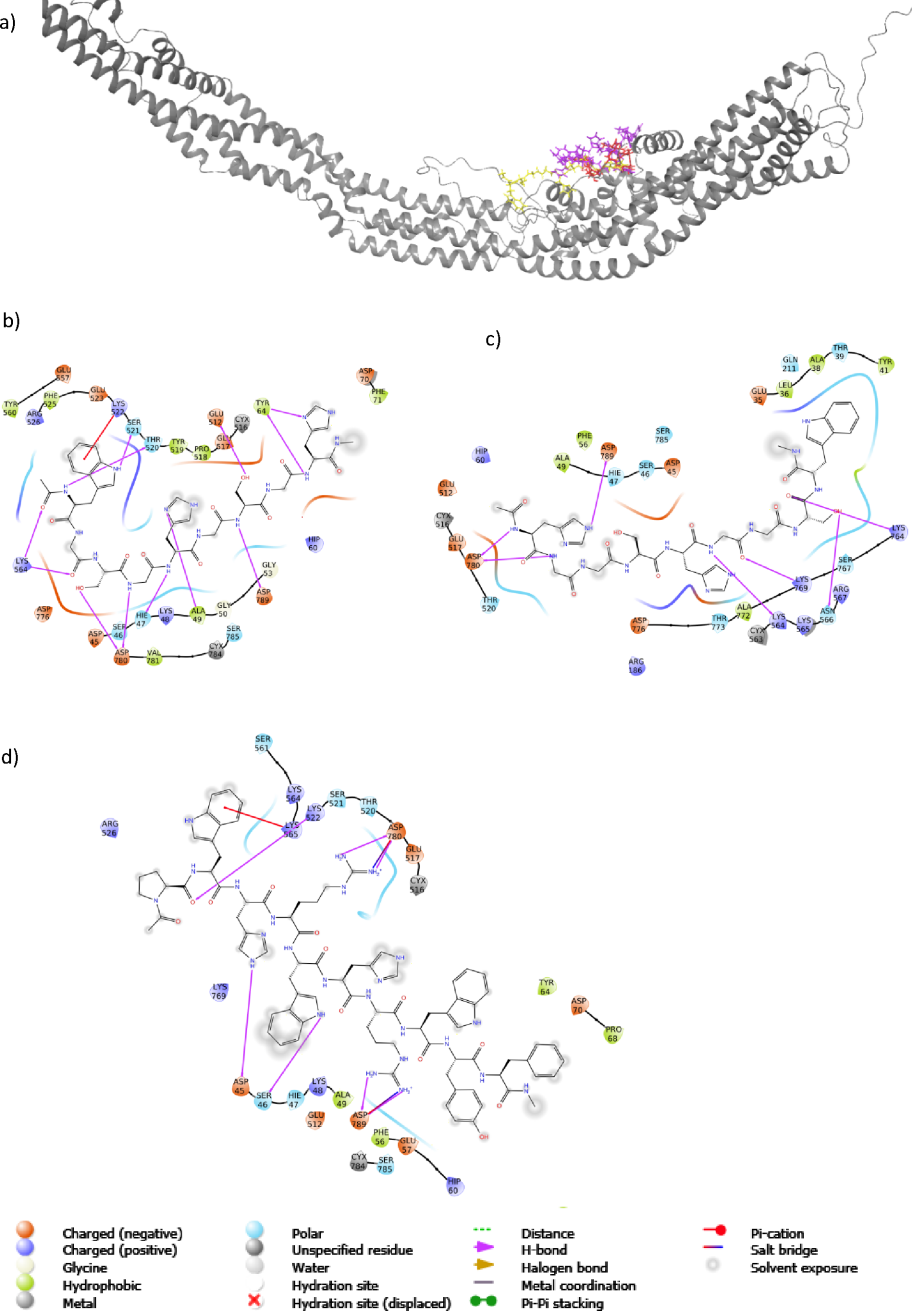
(a) Binding conformations of Peptide A (red), Peptide D (yellow), and Peptide J (purple) on prominin-1, and interactions of the best binding conformations of (b) Peptide A, (c) Peptide D, and (d) Peptide J with prominin-1.

Analysis of interactions revealed (Figure 2) that all three peptides formed multiple hydrogen bond interactions with the receptor, forming multiple interactions with all three extracellular domains. Furthermore, all peptides formed H-bond interactions with ASP780, which also interacted with the amino acid probes at sites 1 and 4. It can also be observed that both peptide A and peptide J formed multiple interactions in CYS516-ARG526, which are located in extracellular loop EC3, which has been identified as a suitable antibody binding site.^32,33^ This suggests that the peptides bind to an accessible site in prominin-1.

### Molecular Dynamics Simulations of Peptides Targeting Prominin-1

The stability of peptide binding to prominin-1 was evaluated using molecular dynamics simulations. The RMSD of the peptides bound to prominin-1 and the ligand–protein contacts during simulation duration are presented in Figure 3.

**Figure 3:**
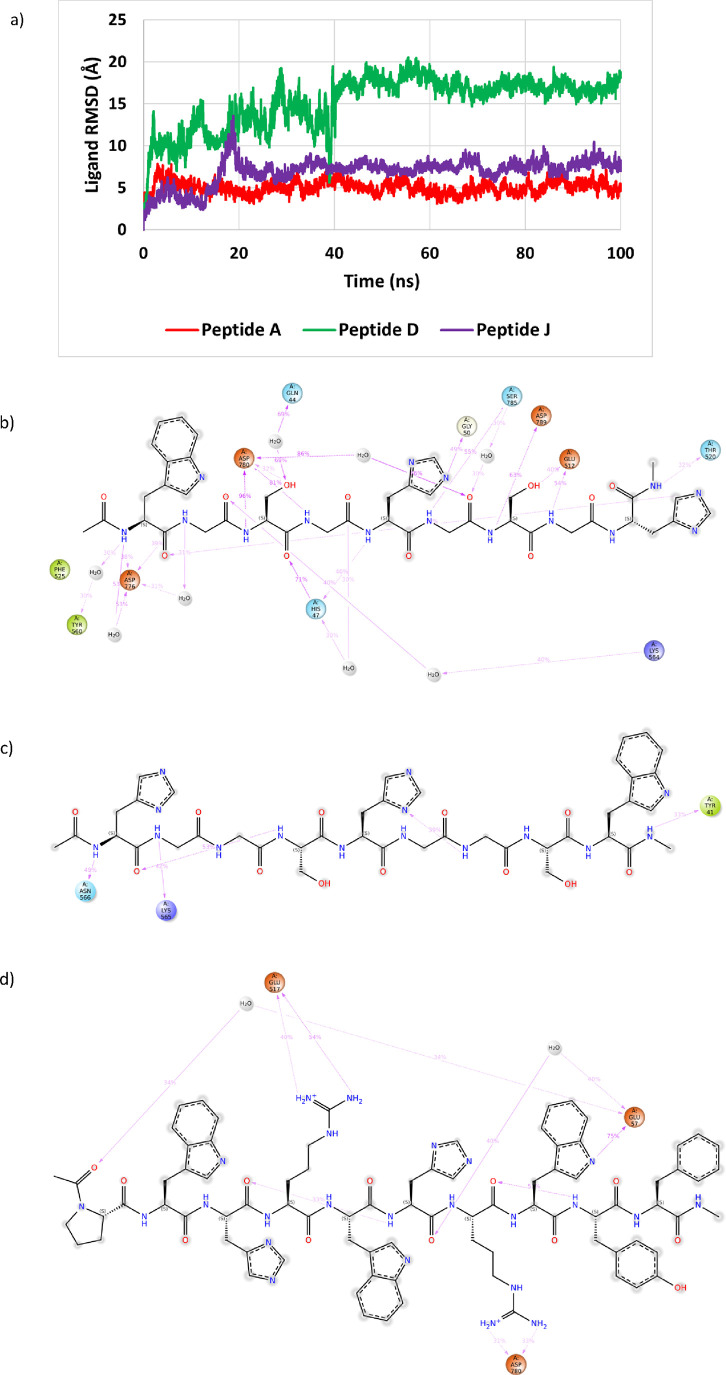
(a) RMSD plots of the peptides bound to prominin-1 and ligand–protein contacts of (b) Peptide A, (c) Peptide D, and (d) Peptide J during MD simulations.

As illustrated in Figure 3a, among the three peptides targeting prominin-1, Peptide A demonstrated the most stable binding, with an average RMSD of 5.5 Å. This also agrees with the interaction diagram (Figure 3b), which shows that Peptide A maintained stable interactions with multiple residues, through more than 50% of the simulation duration. Peptide J also showed moderately stable binding, with an average RMSD of 6.95 Å. In contrast, Peptide D demonstrated unstable binding, with a very high RMSD and a lower number of interactions.

Analysis of binding free energy during the last 50 ns of the simulation is presented in Table 3. Peptide A had the most negative binding energy, indicating a stable binding, while Peptide J also showed a binding free energy less than −50 kcal/mol. For Peptide A, Van der Waal’s interactions were predominant, while Coulombic attractions contributed the most to the binding energy of Peptide J, which can be attributed to the presence of cationic residues in Peptide J. These results suggest that Peptide A and Peptide J can exhibit thermodynamically favorable and stable binding to the predicted binding site of prominin-1. However, since the binding site was predicted computationally instead of being experimentally established, the docking and MD results should be interpreted as a computational basis for candidate prioritization rather than definitive evidence of binding.

**Table 3:** Average MM-GBSA Binding Energies during the Last 50 ns of the Simulation for Prominin-Peptide Complexes

peptide	Δ*G* bind	Δ*G* bind Coulomb	Δ*G* bind covalent	Δ*G* bind H-bond	Δ*G* bind lipophilic	Δ*G* bind solvation	Δ*G* bind VdW	ligand strain energy
Peptide A	–58.028	–29.911	1.182	–3.755	–9.662	53.592	–70.228	38.819
Peptide D	–45.523	–22.844	1.896	–1.455	–9.641	33.444	–45.704	12.664
Peptide J	–51.331	–92.926	1.256	–5.149	–10.062	107.529	–51.147	13.443

### Biolayer Interferometry of Peptides Targeting Prominin-1

The binding kinetics of peptides to prominin-1 protein were analyzed based on the association rate (*K*_a_), dissociation rate (*K*_d_), and binding affinity constant (*K*_D_). Binding parameters and binding curves of the peptides on prominin-1 are given in Table 4 and Figure 4, respectively.

**Table 4:** Association Rate (*K*_a_), Dissociation Rate (*K*_d_), and Affinity Constant (*K*_D_) of the Peptides on Prominin-1^a^

peptide	association rate (K_a_) (1/M s)	dissociation rate (K_d_) (1/s)	affinity constant (K_D_) (M)	*R* ^2^
Peptide A	(2.08 ± 0.63) × 10^1^	(1.40 ± 0.26) × 10^–3^	(6.85 ± 0.82) × 10^–5^	0.877
Peptide D	(7.96 ± 2.39) × 10 °	(6.40 ± 1.26) × 10^–4^	(8.68 ± 4.19) × 10^–5^	0.958
Peptide J	(4.26 ± 3.95) × 10^3^	(1.15 ± 0.35) × 10 °	(4.05 ± 2.94) × 10^–4^	0.948

a The binding parameters are expressed as mean ± standard deviation based on three replicates.

**Figure 4:**
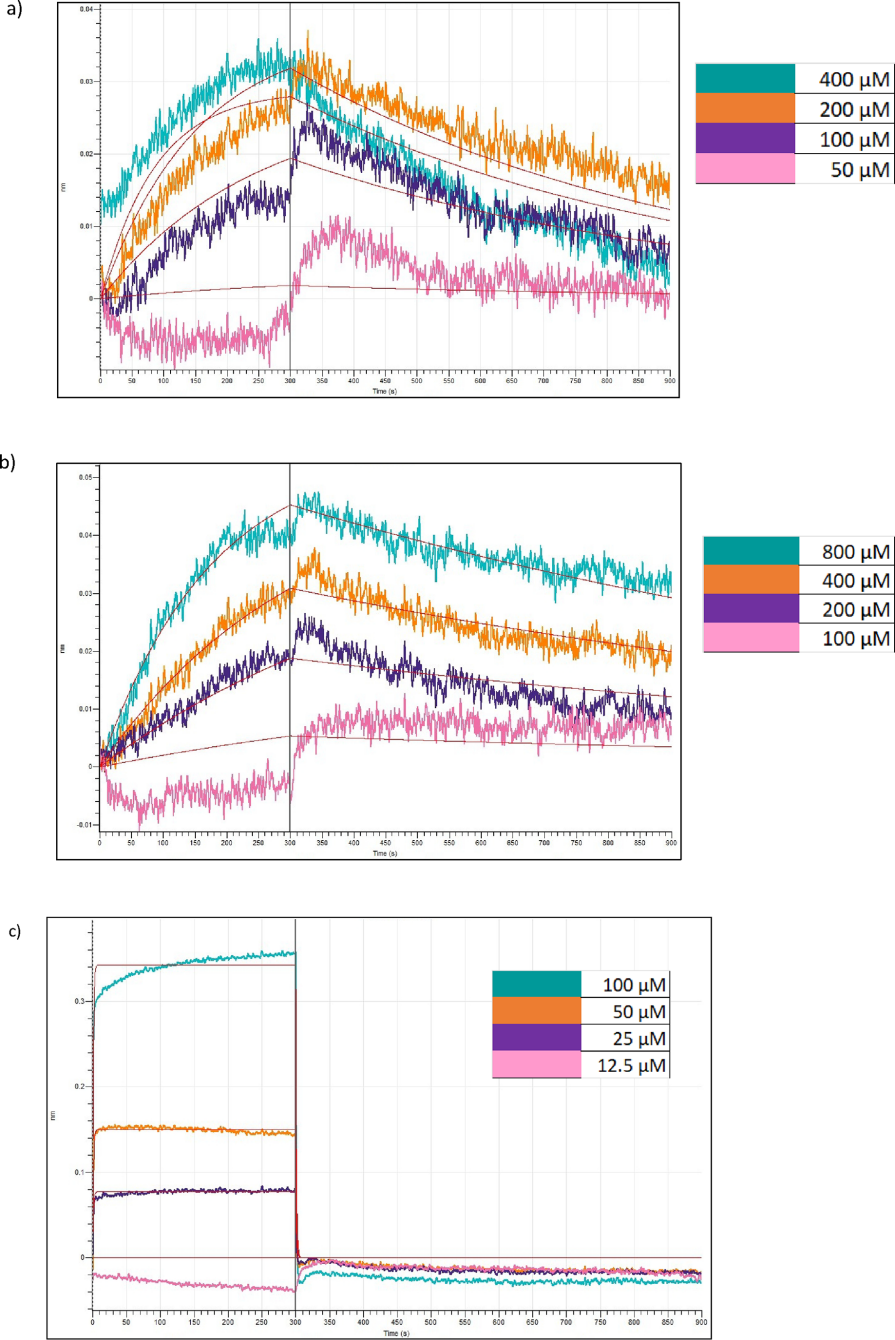
BLI Binding curves of (a) Peptide A, (b) Peptide D, and (c) Peptide J on prominin-1 (5 μg/mL) immobilized on HIS1K biosensors.

Analysis of the fitted curves shows that peptides D and J closely match the experimental data, as reflected by their high *R* ^2^ values. Peptide A exhibits a slightly lower *R* ^2^ but still aligns well with the fitted model at higher concentrations. The deviation observed at the lowest concentration is likely due to a reduced signal-to-noise ratio. Overall, these results support the use of a 1:1 binding model for all three peptides.

As shown in Figure 4, all three peptides showed a dose-dependent binding response on prominin-1, suggesting that all peptides bind to prominin-1 in the tested concentration range. According to Table 4, peptides A and D had mean affinity constants in the micromolar range, while peptide J showed affinity in the low millimolar range. The BLI results for peptides A and D are consistent with the moderate binding suggested by MM-GBSA binding energies.^34^

While Peptide J showed a high association rate, it also showed a much higher dissociation rate than the other two peptides, which can also be observed in the binding curve (Figure 4c). This indicates that peptide J shows less stable binding than the other two peptides, even though it displayed a low MM-GBSA energy and a low RMSD during the MD simulations. Since MM-GBSA energy calculation does not incorporate entropy change, we calculated the entropic contributions using the interaction entropy approach.^27,28^ Peptide J had an entropic penalty of −52.560 kcal/mol, which outweighed the binding energy. In comparison, the entropic penalties for Peptide A (−42.087 kcal/mol) and Peptide D (−22.805 kcal/mol) were not sufficient to overcome the favorable binding energies. This suggests that the low experimental affinity of Peptide J could be a result of entropic contributions. Additionally, analysis of MM-GBSA energy components (Table 4) reveals that Peptide J has a highly positive Δ*G* solvation value. This indicates that the energy required for the desolvation of the receptor and the ligand greatly exceeds the energy released during the solvation of the complex, which could also lead to lower affinity under an aqueous environment.^35^ The unfavorable BLI results for Peptide J suggest that it is not a promising peptide for targeting prominin-1.

Overall, Peptide A performed well in BLI with a comparatively high association rate and a low dissociation rate, while Peptide D also can be considered promising due to a low dissociation rate.

### Peptide Design Targeting Sortilin

To identify the amino acids with the highest predicted affinity to the sortilin active site, the probes were docked into the neurotensin binding site. The docking results of the strongest binding amino acid probes are included in Table 5, and the binding of the amino acids is visualized in Figure 5.

**Table 5:** Docking Scores and Glide Energies of the Strongest Binding Amino Acid Probes on Sortilin^a^

amino acid	docking score (kcal/mol)	Glide energy (kcal/mol)
HIS	–6.266	–28.839
PHE	–4.752	–23.201
TRP	–4.739	–22.367
PRO	–4.457	–17.770

a All values are expressed as the average of the three best conformations.

**Figure 5:**
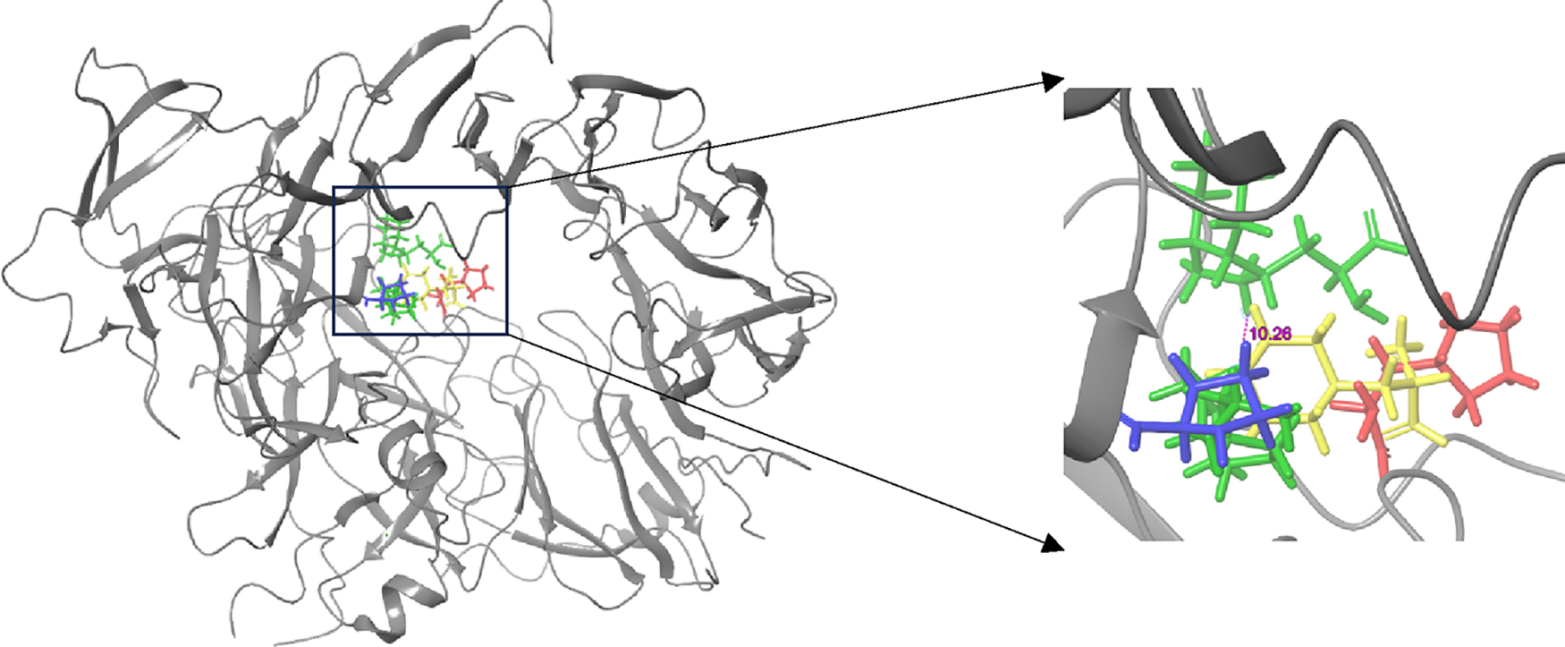
Binding conformations of the strongest binding amino acids at the active site of sortilin: HIS (red), TRP (green), PHE (yellow), and PRO (blue).

According to Table 5, HIS, PHE, TRP, and PRO displayed the strongest binding to the sortilin active site. As illustrated in Figure 5, the probes are docked within close proximity to each other, with a distance of around 2–3 Å between the adjacent probes. It could be observed that the top two conformations for TRP are bound to slightly different locations, with a distance of approximately 10 Å between each other. Based on the binding conformations and affinities of amino acid probes, two peptides were designed by combining these amino acids, also considering their spatial arrangement at the docking site.

The docking results of the peptides on the sortilin active site are presented in Table 6. Among the two peptides, Sortilin_Peptide1 showed slightly stronger binding, with −8.971 kcal/mol. While the docking scores suggested potentially strong binding, the MM-GBSA energies were in the range of −30 to −50 kcal/mol, which is indicative of moderately strong binding.^36–38^

**Table 6:** Docking Results of Sortilin-Targeting Peptides at the Neurotensin Binding Site^a^

peptide	sequence	docking score (kcal/mol)	Glide energy (kcal/mol)	MM-GBSA energy (kcal/mol)
Sortilin_Peptide1	PWFHEHW	–8.971	–81.509	–30.877
Sortilin_Peptide2	PWFHHW	–7.922	–75.525	–34.207

a All values are expressed as the average of the three best conformations

Visualization of peptide binding conformations (Figure 6a) revealed that the peptides bound to the same location as the amino acid probes. According to interaction analysis (Figure 6b,c), both peptides displayed hydrogen bonds, polar, and hydrophobic interactions with multiple residues. Both peptides formed multiple interactions with the ASN373-TYR381 loop, which is reported to be the binding site of several sortilin-binding antibodies.^39^ The peptides also interacted with the LEU321-ASP326 region, which is located in close proximity to PHE317, TYR318, and SER319 residues, which are reported to interact with neurotensin.^40^ This suggests that these two peptides can interact with functionally important residues in sortilin, suggesting that they could be potential preliminary candidates for future biosensing applications.

**Figure 6:**
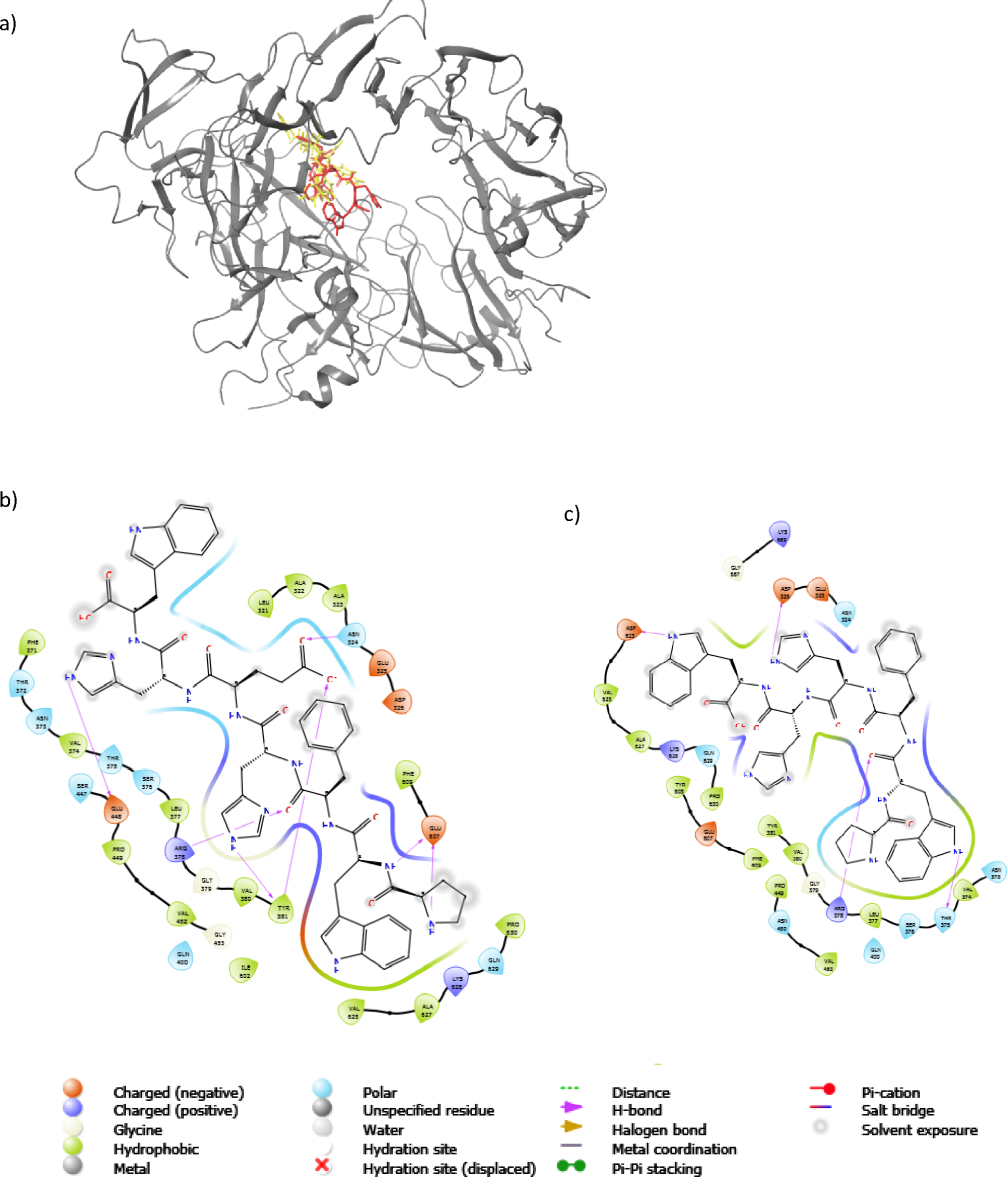
(a) Binding conformations of Sortilin_Peptide1 (red) and Sortilin_Peptide2 (yellow) at the active site of sortilin, and the interactions of (b) Sortilin_Peptide1 and (c) Sortilin_Peptide2 with sortilin.

### Molecular Dynamics Simulations of Peptides Targeting Sortilin

Molecular dynamics simulations revealed that Sortilin_Peptide2 demonstrated more stable binding to sortilin compared to Sortilin_Peptide2, with an average RMSD of 7.90 Å (Figure 7a). This also agrees with the interaction analysis, which displays that Sortilin_Peptide2 formed a number of interactions with interaction fractions over 50% (Figure 7b). In comparison, Sortilin_Peptide2 demonstrated less stable binding, with high RMSD and a lower number of stable interactions.

**Figure 7:**
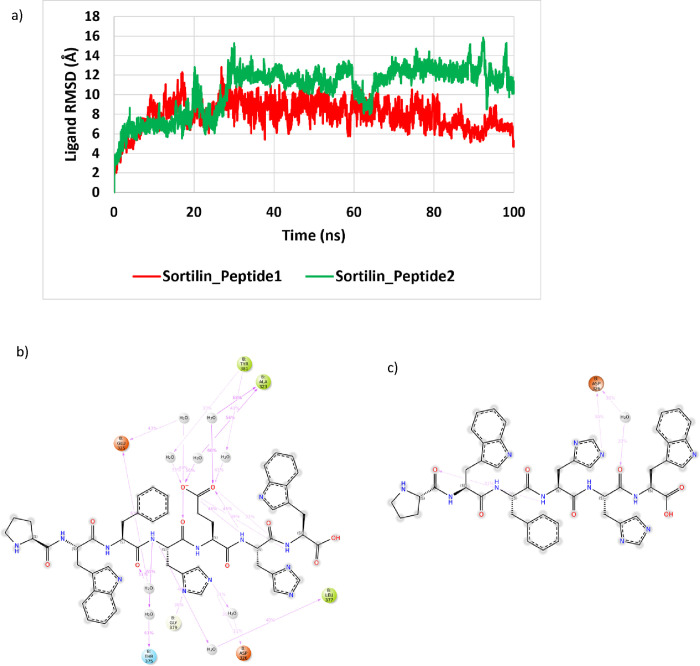
(a) RMSD plots for the peptides bound to sortilin, and ligand–protein contacts for (b) Sortilin_Peptide1 and (c) Sortilin_Peptide2 with sortilin during MD simulations.

Analysis of binding free energies during the simulation (Table 7) revealed that both peptides showed moderately stable binding to sortilin, with overall binding energies around −40 kcal/mol. Sortilin_Peptide1 demonstrated stronger binding, which also agrees with the higher number of interactions as shown in Figure 7. Van der Waal’s interactions were predominant for both peptides.

**Table 7:** Average MM-GBSA Binding Energies during the Last 50 ns of the Simulation for Sortilin-Peptide Complexes

peptide	Δ*G* bind	Δ*G* bind Coulomb	Δ*G* bind covalent	Δ*G* bind H-bond	Δ*G* bind lipophilic	Δ*G* bind solvation	Δ*G* bind VdW	ligand strain energy
Sortilin_Peptide1	–40.769	75.122	4.879	–2.120	–13.342	–47.285	–55.895	14.862
Sortilin_Peptide2	–36.155	–18.170	2.286	–1.387	–10.686	29.394	–34.730	9.782

### Biolayer Interferometry of Peptides Targeting Sortilin

The binding kinetics of peptides to sortilin were analyzed in vitro using BLI, based on the association rate (*K*_a_), dissociation rate (*K*_d_), and binding affinity constant (*K*_D_). Binding parameters and binding curves of the peptides on sortilin are given in Table 8 and Figure 8, respectively.

**Table 8:** Association Rate (*K*_a_), Dissociation Rate (*K*_d_), and Affinity Constant (*K*_D_) of the Peptides on Sortilin^a^

using 1:1 binding model				
**peptide**	**association rate (K**_**a**_ **)**(1/M s)	**dissociation rate (K**_**d**_ **)**(1/s)	**affinity constant (K**_**D**_ **) (M)**	**R** ^**2**^
Sortilin_Peptide1	(3.18 ± 2.09) × 10^3^	(3.26 ± 0.84) × 10^–3^	(3.74 ± 3.12) × 10^–6^	0.829
Sortilin_Peptide2	(1.91 ± 2.69) × 10^3^	(5.62 ± 1.01 × 10^–3^	(2.91 ± 4.18) × 10^–5^	0.852

**using heterogeneous binding model**							
**peptide**	**K**_**a**_(1/M s)	**K**_**a2**_(1/M s)	**(K**_**d**_ **)**(1/s)	**(K**_**d2**_ **)**(1/s)	**(K**_**D**_ **) (M)**	**(K**_**D2**_ **) (M)**	**R** ^**2**^
Sortilin_Peptide1	(4.39 ± 3.78) × 10^2^	(1.07 ± 1.01) × 10^4^	(4.21 ± 1.06) × 10^–7^	(4.11 ± 5.09) × 10^–2^	(1.58 ± 1.10) × 10^–9^	(7.27 ± 6.85) × 10^–6^	0.974
Sortilin_Peptide2	(2.21 ± 1.85) × 10^2^	(3.12 ± 5.33) × 10^4^	(1.04 ± 0.94) × 10^–3^	(4.85 ± 2.73) × 10^–2^	(4.38 ± 7.07) × 10^–5^	(4.32 ± 6.97) × 10^–4^	0.958

a The binding parameters are expressed as mean ± standard deviation based on three replicates.

**Figure 8:**
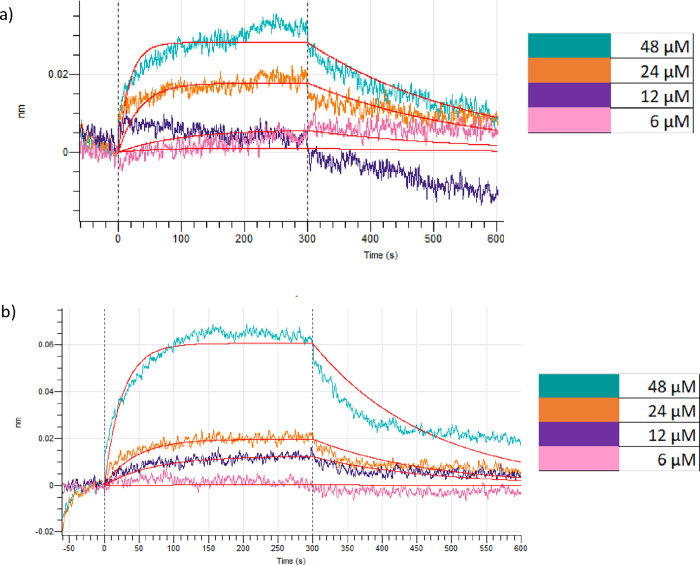
BLI Binding curves of (a) Sortilin_Peptide1 and (b) Sortilin_Peptide2 on sortilin (10 μg/mL) immobilized on HIS1K biosensors.

Figure 8 shows that both peptides have shown a dose-dependent response in the association step, showing that both peptides bind to sortilin in the tested concentration ranges. As shown in Table 8, when the 1:1 binding model was used, both peptides had mean affinities in the micromolar range, with Sortilin_Peptide1 showing significantly stronger affinity, as shown by the smaller affinity constant. Sortilin_Peptide1 showed a higher association rate and lower dissociation rate than Sortilin_Peptide2, suggesting that it is more likely to bind with sortilin and also less likely to dissociate. It can also be observed that Sortilin_Peptide2 showed higher deviation in kinetic parameters, which suggests inconsistent binding.

The *R* ^2^ values for these two peptides are lower compared to those of prominin-1-targeting peptides, and some of the curves exhibited plateauing at non-zero concentrations, which could be an indication of heterogeneous binding. Therefore, the BLI curves were also fitted to a heterogeneous binding model. For Sortilin_Peptide1, the heterogeneous binding model yielded two binding components: a nanomolar-level affinity component with very slow dissociation and a weaker micromolar-level component. However, the nanomolar-level affinity predicted by this model is not consistent with the observed binding behavior and may reflect a surface-associated or irreversible binding contribution rather than the true binding affinity. The micromolar-level affinity component is consistent with the results from the 1:1 model fitting. For Sortilin_Peptide2, both components were in the micromolar range, consistent with the results from 1:1 model fitting.

Overall, the BLI results agree with MD simulations, which revealed that Sortilin_Peptide1 demonstrated more stable binding than Sortilin_Peptide2, as evidenced by lower RMSD, more negative MM-GBSA binding energy, and higher consistency of interactions. Therefore, BLI results suggest that Sortilin_Peptide1 is a better candidate for targeting sortilin than Sortilin_Peptide2.

A possible limitation in the BLI experiments is that the use of Anti-Penta-HIS biosensors could cause surface-related artifacts due to the interaction between the sensor surface and histidine residues in the peptides. Therefore, validation of these BLI results using orthogonal immobilization strategies will be a focus in future studies.

## Conclusions

In this study, we utilized a peptide design approach based on residue-level binding to design peptide binders targeting two clinically significant biomarkers, prominin-1 and sortilin. Through a systematic approach consisting of molecular docking, molecular dynamics simulations, and MM-GBSA analysis, we identified peptide candidates that were predicted to engage in stable binding to functionally relevant regions of the two receptors. Experimental validation using BLI revealed micromolar levels of binding to the receptors. While the heterogeneous binding model for sortilin-binding peptides suggested a potential nanomolar-level affinity constant, it was likely a result of surface-related artifacts or irreversible binding rather than the true binding affinity. The overall binding behavior of all peptides was consistent with micromolar affinities. These findings suggest that these peptides may serve as preliminary candidates for future biosensing applications.

This work demonstrates the potential of this approach for developing peptides that target clinically significant biomarkers. Future work will focus on improving the affinity and specificity of peptides to the selected biomarkers, experimentally elucidating the binding modes and functional implications of the peptides on the biomarkers, and evaluating their effectiveness in relevant in vitro and in vivo models. Overall, this approach provides a foundation for future studies of molecular targeting based on residue-level binding.

## Supplementary Material



## Data Availability

All relevant data are included in the manuscript and supplementary data. Molecular dynamic trajectories for protein-peptide complexes can be found in this Zenodo repository, https://zenodo.org/records/19857695. Any additional information is available from the authors upon request.

## Notes

SiteMap predicted druggable sites for Prominin-1 (Figure S1); properties of predicted druggable sites of Prominin-1 (Table S1); docking results for all peptides designed targeting Prominin-1 (Table S2); interactions between the peptides and Prominin-1 (Figure S2); and trajectory videos for MD simulations (https://zenodo.org/records/19857695) (PDF)

## #

University of Massachusetts Amherst, Amherst, MA 01003, United States (N.M.)

## References

[1] Passaro A., Al Bakir M., Hamilton E. G., Diehn M., André F., Roy-Chowdhuri S., Mountzios G., Wistuba I. I., Swanton C., Peters S. (2024). Cancer biomarkers: Emerging trends and clinical implications
for personalized treatment. Cell.

[2] Wang X., Li B., Li R., Yang Y., Zhang H., Tian B., Cui L., Weng H., Wei F. (2018). Anti-CD133 monoclonal antibody conjugated
immunomagnetic nanosensor for molecular imaging of targeted cancer
stem cells. Sens. Actuators, B.

[3] Barzegar
Behrooz A., Syahir A., Ahmad S. (2019). CD133: beyond a cancer
stem cell biomarker. J. Drug Target.

[4] Ding J., Xu W., Tan J., Liu Z., Huang G., Wang S., He Z. (2022). Fluorescence detection
of cancer stem cell markers using a sensitive
nano-aptamer sensor. Frontiers in Chemistry.

[5] Pleskač P., Fargeas C. A., Veselska R., Corbeil D., Skoda J. (2024). Emerging roles
of prominin-1 (CD133) in the dynamics of plasma membrane architecture
and cell signaling pathways in health and disease. Cell. Mol. Biol. Lett..

[6] Fathi F., Rahbarghazi R., Movassaghpour A. A., Rashidi M.-R. (2019). Detection of CD133-marked
cancer stem cells by surface plasmon resonance: Its application in
leukemia patients. Biochimica et Biophysica
Acta (BBA) - General Subjects.

[7] Gisina A., Yarygin K., Lupatov A. (2024). The Impact
of Glycosylation on the
Functional Activity of CD133 and the Accuracy of Its Immunodetection. Biology (Basel).

[8] Glumac P. M., LeBeau A. M. (2018). The role of CD133
in cancer: a concise review. Clin. Transl. Med..

[9] Bidlingmaier S., Zhu X., Liu B. (2008). The utility
and limitations of glycosylated human CD133
epitopes in defining cancer stem cells. J. Mol.
Med. (Berl).

[10] Swaminathan S. K., Olin M. R., Forster C. L., Cruz K. S. S., Panyam J., Ohlfest J. R. (2010). Identification of a novel monoclonal
antibody recognizing
CD133. Journal of Immunological Methods.

[11] Chenaghlou S., Khataee A., Jalili R., Rashidi M.-R., Khalilzadeh B., Woo Joo S. (2021). Gold nanostar-enhanced electrochemiluminescence immunosensor
for highly sensitive detection of cancer stem cells using CD133 membrane
biomarker. Bioelectrochemistry.

[12] Shigdar S., Qiao L., Zhou S.-F., Xiang D., Wang T., Li Y., Lim L. Y., Kong L., Li L., Duan W. (2013). RNA aptamers
targeting cancer stem cell marker CD133. Cancer
Letters.

[13] Sun J., Zhang C., Liu G., Liu H., Zhou C., Lu Y., Zhou C., Yuan L., Li X. (2012). A novel mouse CD133
binding-peptide screened by phage display inhibits cancer cell motility
in vitro. Clinical & Experimental Metastasis.

[14] Marsland M., Dowdell A., Faulkner S., Gedye C., Lynam J., Griffin C. P., Marsland J., Jiang C. C., Hondermarck H. (2023). The Membrane
Protein Sortilin Is a Potential Biomarker and Target for Glioblastoma. Cancers (Basel).

[15] Mitok K. A., Keller M. P., Attie A. D. (2022). Sorting
through the extensive and
confusing roles of sortilin in metabolic disease. J. Lipid Res..

[16] Herda S., Raczkowski F., Mittrücker H.
W., Willimsky G., Gerlach K., Kühl A. A., Breiderhoff T., Willnow T. E., Dörken B., Höpken U. E., Rehm A. (2012). The sorting receptor Sortilin exhibits a dual function in exocytic
trafficking of interferon-γ and granzyme A in T cells. Immunity.

[17] Vaegter C. B., Jansen P., Fjorback A. W., Glerup S., Skeldal S., Kjolby M., Richner M., Erdmann B., Nyengaard J. R., Tessarollo L., Lewin G. R., Willnow T. E., Chao M. V., Nykjaer A. (2011). Sortilin associates with Trk receptors
to enhance anterograde
transport and neurotrophin signaling. Nat. Neurosci.

[18] Mo̷ller P. L., Rohde P. D., Winther S., Breining P., Nissen L., Nykjaer A., Bo̷ttcher M., Nyegaard M., Kjolby M. (2021). Sortilin as
a Biomarker for Cardiovascular Disease Revisited. Front. Cardiovasc. Med..

[19] Oh T. J., Ahn C. H., Kim B.-R., Kim K. M., Moon J. H., Lim S., Park K. S., Lim C., Jang H., Choi S. H. (2017). Circulating
sortilin level as a potential biomarker for coronary atherosclerosis
and diabetes mellitus. Cardiovasc. Diabetol..

[20] Roselli S., Pundavela J., Demont Y., Faulkner S., Keene S., Attia J., Jiang C. C., Zhang X. D., Walker M. M., Hondermarck H. (2015). Sortilin is
associated with breast cancer aggressiveness
and contributes to tumor cell adhesion and invasion. Oncotarget.

[21] Farahi L., Ghaemimanesh F., Milani S., Razavi S. M., Akhondi M. M., Rabbani H. (2019). Sortilin as
a Novel Diagnostic and Therapeutic Biomarker
in Chronic Lymphocytic Leukemia. Avicenna J.
Med. Biotechnol..

[22] Ek M., Nilvebrant J., Nygren P. Å., Ståhl S., Lindberg H., Löfblom J. (2024). An anti-sortilin affibody-peptide
fusion inhibits sortilin-mediated progranulin degradation. Front. Immunol..

[23] Jumper J., Evans R., Pritzel A., Green T., Figurnov M., Ronneberger O., Tunyasuvunakool K., Bates R., Žídek A., Potapenko A., Bridgland A., Meyer C., Kohl S. A. A., Ballard A. J., Cowie A., Romera-Paredes B., Nikolov S., Jain R., Adler J., Back T., Petersen S., Reiman D., Clancy E., Zielinski M., Steinegger M., Pacholska M., Berghammer T., Bodenstein S., Silver D., Vinyals O., Senior A. W., Kavukcuoglu K., Kohli P., Hassabis D. (2021). Highly accurate protein
structure prediction with AlphaFold. Nature.

[24] Leloup N., Lössl P., Meijer D. H., Brennich M., Heck A. J. R., Thies-Weesie D. M. E., Janssen B. J. C. (2017). Low pH-induced
conformational change and dimerization of sortilin triggers endocytosed
ligand release. Nat. Commun..

[25] Sterling T., Irwin J. J. (2015). ZINC 15 – Ligand Discovery
for Everyone. J. Chem. Inf. Model..

[26] Mallawarachchi S., Irigoyen S., Mandadi K., Borneman J., Fernando S. (2026). Design of
Highly Specific Antimicrobial Peptides Targeting the BamA protein
of Candidatus Liberibacter asiaticus. ACS Omega.

[27] Duan L., Liu X., Zhang J. Z. H. (2016). Interaction Entropy: A New Paradigm for Highly Efficient
and Reliable Computation of Protein–Ligand Binding Free Energy. J. Am. Chem. Soc..

[28] Ekberg V., Ryde U. (2021). On the Use of Interaction
Entropy and Related Methods to Estimate
Binding Entropies. J. Chem. Theory Comput..

[29] Shmelkov S. V., St.Clair R., Lyden D., Rafii S. (2005). AC133/CD133/Prominin-1. Int. J. Biochem. Cell Biol..

[30] Reddy
Chichili V. P., Kumar V., Sivaraman J. (2013). Linkers in
the structural biology of protein-protein interactions. Protein Sci..

[31] Wang H., Mulgaonkar N., Mallawarachchi S., Ramasamy M., Padilla C. S., Irigoyen S., Coaker G., Mandadi K. K., Fernando S. (2022). Evaluation
of Candidatus Liberibacter Asiaticus Efflux Pump Inhibition by Antimicrobial
Peptides. Molecules.

[32] Wang D., Guo Y., Li Y., Li W., Zheng X., Xia H., Mao Q. (2015). Detection of CD133
expression in U87 glioblastoma cells using a novel
anti-CD133 monoclonal antibody. Oncol Lett..

[33] Ghani S., Yarian F., Bandehpour M., Kazemi B. (2021). An In-silico Approach
and Experimental Analysis Combination: Two Strategies for Selecting
the third Extracellular Domain (D-EC3) of Human CD133 Marker as a
Target for Detection of Cancer Stem Cells. Iran
J. Pharm. Res..

[34] Mulgaonkar N., Wang H., Mallawarachchi S., Růžek D., Martina B., Fernando S. (2023). In silico and in vitro evaluation
of imatinib as an inhibitor for SARS-CoV-2. J. Biomol. Struct. Dyn..

[35] Mallawarachchi S., Nangia A., Ibrahim M. J., Haq A., Fernando S., King M. D. (2026). Evaluation of molecular interactions
of vaping juice
components with ACE2 receptor. Sci. Rep..

[36] Wang E., Sun H., Wang J., Wang Z., Liu H., Zhang J. Z., Hou T. (2019). End-point
binding free energy calculation with MM/PBSA and MM/GBSA:
strategies and applications in drug design. Chem. Rev..

[37] Genheden S., Ryde U. (2015). The MM/PBSA and MM/GBSA methods to
estimate ligand-binding affinities. Expert Opinion
on Drug Discovery.

[38] Haq A., Mallawarachchi S., Anderson A., Khaleghi L., Manujitha L., Fernando S. (2025). In Silico Evaluation of Potential
Hit Molecules Against
Multiple Serotypes of Dengue Virus Envelope Glycoprotein. Molecules.

[39] Rosenthal, A. S. , Michael, T. K. Anti - Sortilin Antibodies and Methods of use thereof. US010428150B2, 2019.

[40] Quistgaard E. M., Gro̷ftehauge M. K., Madsen P., Pallesen L. T., Christensen B., So̷rensen E. S., Nissen P., Petersen C. M., Thirup S. S. (2014). Revisiting
the structure of the Vps10 domain of human
sortilin and its interaction with neurotensin. Protein Sci..

