# Supplementary material for: Rational Design of Peptide Binders Targeting Prominin‑1 and Sortilin for Molecular Sensing Applications
Source: ACS Omega. 2026 Jul 1;11(27):39922–34. doi: 10.1021/acsomega.6c01105 (PMC13382844; doi:10.1021/acsomega.6c01105)
Supplement: Supplementary file 1 [file ao6c01105_si_001.pdf]

Rational Design of Peptide Binders Targeting Prominin-1 and Sortilin for Molecular Sensing Applications

Samavath Mallawarachchi[1], Nirmitee Mulgaonkar[1,#], Samuel Mabbott[2], Shreya Raghavan[2] and Sandun Fernando[1,*]

[1] Department of Biological and Agricultural Engineering, Texas A&M University, College Station, TX 77843
[2] Department of Biomedical Engineering, Texas A&M University, College Station, TX 77843

* Corresponding author's email address: sfernando@tamu.edu

[#] Current address of this author: University of Massachusetts Amherst, Amherst, MA 01003

[#] Current address of this author: University of Massachusetts Amherst, Amherst, MA 01003

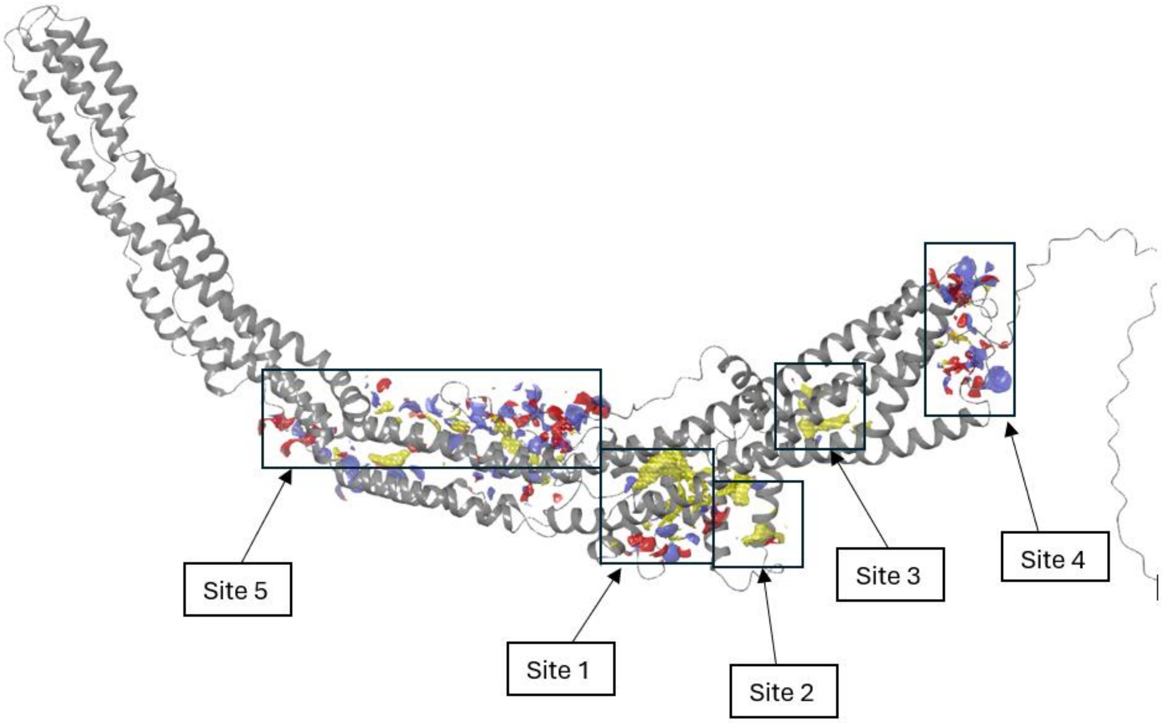

Figure S1. Potential druggable sites for Prominin-1 predicted by SiteMap. Hydrogen-bond donors, hydrogen-bond acceptors and hydrophobic regions are indicated in blue, red and yellow, respectively.

Table S1. Properties of the potential druggable sites predicted by SiteMap. Site score and druggability score indicate the suitability of the site of ligand and drug-type ligand binding, respectively. Exposure and enclosure are measures of how open a site is to the solvent, with low exposure values and high enclosure values indicating clear binding pockets.

Site	Site Score	Druggability (D) Score	Exposure	Enclosure	Site volume (Å^3)	Donor/ acceptor ratio	Hydrophobic/ hydrophilic ratio
1	1.179	1.295	0.507	0.751	471.2	1.774	9.666
2	1.14	1.247	0.541	0.735	263.7	1.005	8.399
3	1.094	1.201	0.507	0.748	180.7	2.522	35.519
4	0.994	0.943	0.634	0.688	302.8	0.532	0.175
5	0.993	1.05	0.638	0.618	950.7	0.876	0.788

Table S2. Docking scores, Glide energies and interactions of all the peptides on prominin-1. Docking scores and energies are expressed as the average of the three best conformations.

Peptide	Docking score	Glide energy	H-Bond and pi-cation interactions
Peptide A	-8.527	-84.700	HIE47, ALA49, TYR64, GLU512, THR520, SER521, LYS522, LYS564, ASP780, ASP789
Peptide B	-8.226	-72.955	ASP45, HIE47, SER521, LYS522, ARG526, LYS564, ASP780, ASP789
Peptide C	-8.790	-77.875	ASP45, HIE47, TYR64, GLU512, GLU517, LYS564, ASP780
Peptide D	-8.508	-81.450	LYS564, ASN566, LYS764, LYS769, ASP780, ASP789
Peptide E	-8.237	-72.080	LYS48, GLU57, GLU523, LYS564, ASP780
Peptide F	-8.860	-75.854	TYR41, ASP45, SER46, GLY53, HIP60, TYR64, GLU512, THR520, LYS564
Peptide G	-9.205	-78.034	ASP45, HIE47, ASP70, GLU523, LYS564
Peptide H	-8.467	-74.272	ALA49, GLU512, THR520, LYS522, LYS564, ASP789
Peptide I	-8.168	-63.438	ASP70, GLU512, LYS564, ASP780
Peptide J	-8.939	-71.476	ASP45, SER46, LYS522, LYS565, ASP780, ASP789

Peptide A

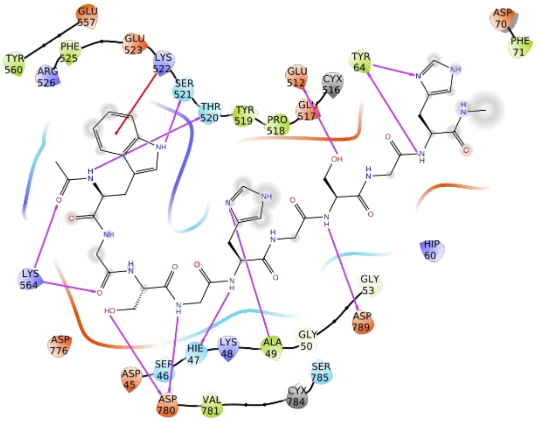

Peptide B

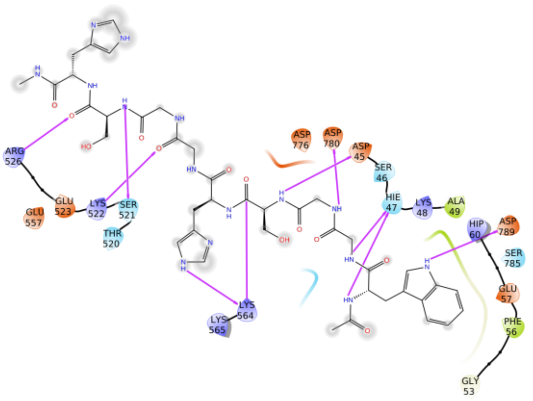

Peptide C

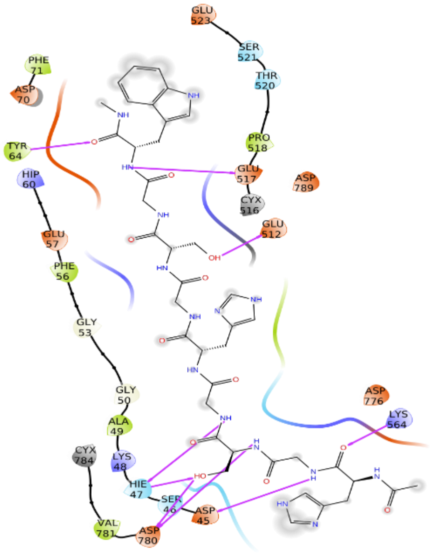

Peptide D

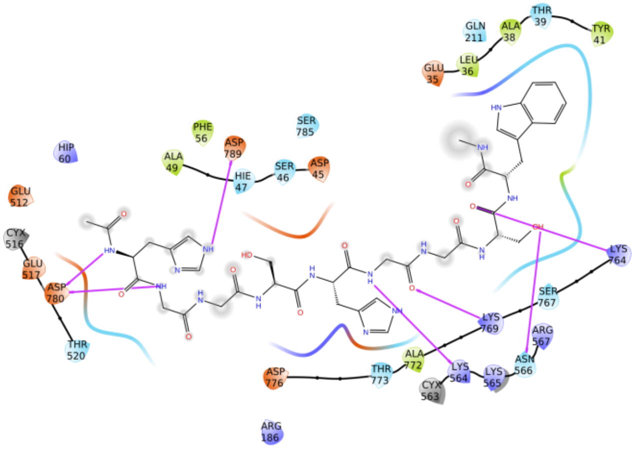

Peptide E

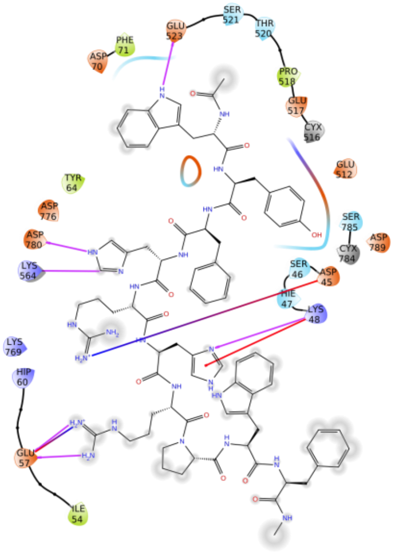

Peptide F

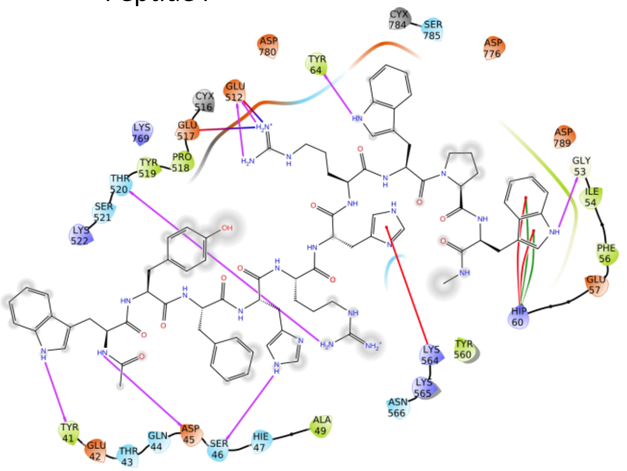

Peptide G

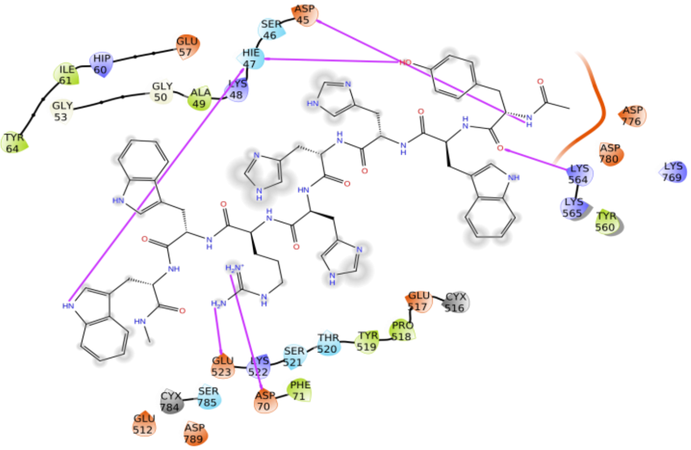

Peptide H

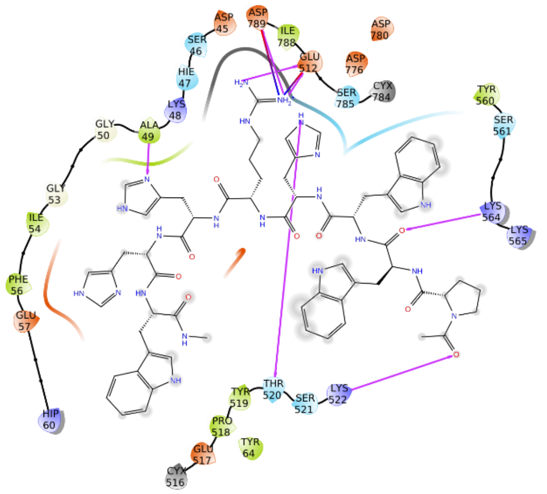

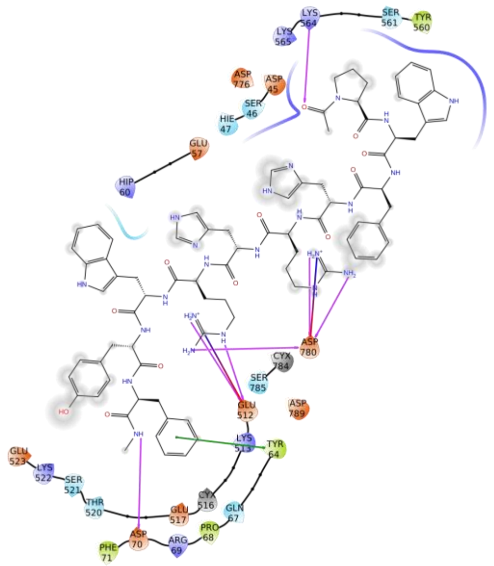

Peptide J

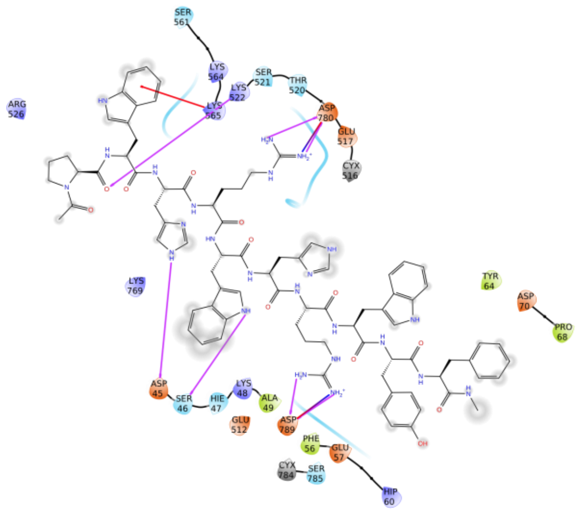

Figure S2. Interactions of the best binding conformation of all peptides targeting prominin-1.

Trajectory videos

Molecular dynamic trajectories for protein-peptide complexes can be found in this Zenodo repository.

https://zenodo.org/records/19857695
